# Notable challenges posed by long-read sequencing for the study of transcriptional diversity and genome annotation

**DOI:** 10.1101/gr.279865.124

**Published:** 2025-04

**Authors:** Carolina Monzó, Adam Frankish, Ana Conesa

**Affiliations:** 1 Institute for Integrative Systems Biology (I2SysBio), Spanish National Research Council (CSIC), Paterna 46980, Spain;; 2 European Molecular Biology Laboratory, European Bioinformatics Institute, Wellcome Genome Campus Hinxton, Cambridge CB10 1SA, United Kingdom

## Abstract

Long-read sequencing (LRS) technologies have revolutionized transcriptomic research by enabling the comprehensive sequencing of full-length transcripts. Using these technologies, researchers have reported tens of thousands of novel transcripts, even in well-annotated genomes, while developing new algorithms and experimental approaches to handle the noisy data. The Long-read RNA-seq Genome Annotation Assessment Project community effort benchmarked LRS methods in transcriptomics and validated many novel, lowly expressed, often times sample-specific transcripts identified by long reads. These molecules represent deviations of the major transcriptional program that were overlooked by short-read sequencing methods but are now captured by the full-length, single-molecule approach. This Perspective discusses the challenges and opportunities associated with LRS’ capacity to unravel this fraction of the transcriptome, in terms of both transcriptome biology and genome annotation. For transcriptome biology, we need to develop novel experimental and computational methods to effectively differentiate technology errors from rare but real molecules. For genome annotation, we must agree on the strategy to capture molecular variability while still defining reference annotations that are useful for the genomics community.

Long-read sequencing (LRS) technologies, such as those developed by Pacific Biosciences (PacBio) and Oxford Nanopore Technologies (ONT), have revolutionized genomic and transcriptomic research. Their ability to generate very long reads has enabled significant advancements, including complete sequencing of human chromosomes (Nurk et al. 2022) and full-length sequencing of single-molecule transcripts spanning kilobases (Sharon et al. 2013; Weirather et al. 2017; Soneson et al. 2019). This unprecedented capability earned LRS recognition by Nature Methods as the Method of the Year in 2022, highlighting its transformative impact on both fields (Marx 2023). One of the most significant contributions of long-read methods to the study of transcription is their capacity to uncover alternative isoforms with a confidence not present in short-read methods, which has led to the discovery of tens of thousands of novel transcripts even in well-annotated organisms (Roach et al. 2020; Glinos et al. 2022; Veiga et al. 2022; Zhang et al. 2022), and represents a data source of great value for the de novo annotation of the Earth BioGenome Project (Lawniczak et al. 2022). Despite its strengths, LRS presents several shortcomings. The quality of long-read RNA sequencing (lrRNA-seq) can be compromised by factors such as RNA degradation, biases introduced during library preparation, sequencing errors, and inaccurate bioinformatic processing during mapping, transcript assembly, and quantification, which may lead to the incorrect identification of transcript models, i.e., computational representations of transcripts depicting their transcription start and termination sites (TSS and TTS) and intron composition (Amarasinghe et al. 2020; Marx 2023). Most lrRNA-seq experiments rely on cDNA libraries, as they provide high sequencing throughput and accuracy. However, reverse transcription may introduce errors driven by specific sequences present in the RNA's primary sequence. These sequences can promote single-nucleotide errors and mispriming, resulting in faulty cDNA molecules (technical artifacts) that inaccurately represent structural variations (Verwilt et al. 2023). The ONT direct RNA-seq method can potentially overcome these issues while also identifying RNA modifications in the native molecule. However, sequencing throughput from current direct RNA protocols is still relatively low compared to cDNA-based protocols (∼20 M reads in direct RNA protocols [Oxford Nanopore Technologies 2019] vs. ∼130 M reads in cDNA-based protocols [Aguzzoli Heberle et al. 2024]), which compromises transcript identification. Despite these shortcomings, direct RNA holds great potential for improving transcript identification in the future.

A significant challenge in the analysis of lrRNA-seq data is the accurate identification of novel transcripts while effectively distinguishing them from artifacts introduced by the technology. To address this, various software tools have been created for reconstructing transcript models from LRS, and recently the technology has been subjected to rigorous benchmarking (Križanovic et al. 2018; Soneson et al. 2019; Kuo et al. 2020; Dong et al. 2023; Su et al. 2024; Pardo-Palacios et al. 2024b). The most comprehensive study to evaluate lrRNA-seq methods to date is the Long-read RNA-seq Genome Annotation Assessment Project (LRGASP), a community effort aimed at systematically evaluating library preparation, sequencing platforms, and analysis tools for the identification and quantification of transcripts using LRS technologies (Pardo-Palacios et al. 2024b).

LRGASP included sequencing from PacBio, ONT, and Illumina short reads, four library preparation methods, and the use of SQANTI3, a common tool for LRS quality control, to evaluate ∼50 analysis pipelines (Pardo-Palacios et al. 2024a). In this scheme, full-splice-match (FSM) transcripts align with reference transcripts at all splice junctions, incomplete-splice-match (ISM) transcripts lack one or more junctions at the 5′ or 3′ ends, which could indicate RNA degradation or alternative initiation/termination sites, novel-in-catalog (NIC) transcripts exhibit new combinations of splice sites, and novel-not-in-catalog (NNC) transcripts have at least one novel donor or acceptor site (Fig. 1A; Tardaguila et al. 2018).

**Figure 1.**
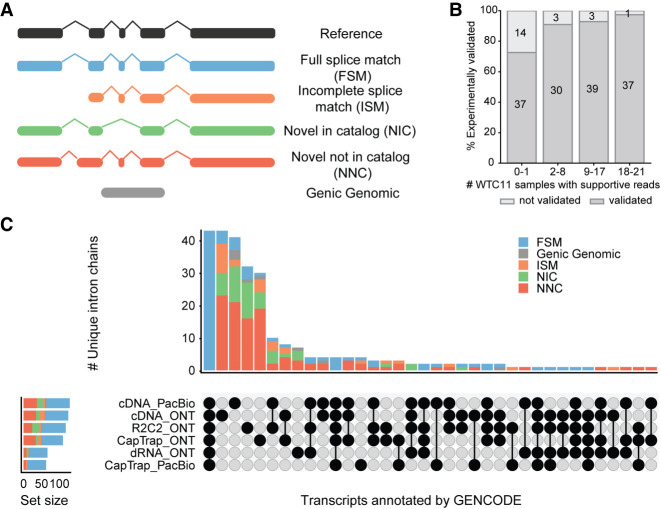
LRGASP identifies many transcripts only expressed in one or a few samples. (*A*) Main SQANTI3 structural categories for transcript models of known genes. (*B*) Fraction of experimentally validated transcripts as a function of the number of WTC11 samples in which supportive reads were observed. (*C*) Structural category classification and intersection of 50 loci from the WTC11 samples that were not identified by any transcript identification tool but were manually annotated by GENCODE. These loci were selected for having mapped reads across all six library preparation and sequencing platform combinations. (*B*,*C*) Adapted from Pardo-Palacios et al. (2024b).

The LRGASP uncovered significant discrepancies among lrRNA-seq methods, particularly in the number and identity of transcripts in the novel SQANTI3 structural categories (ISM, NIC, and NNC), as well as the degree of support from orthogonal data (Pardo-Palacios et al. 2024b). The authors concluded that these discrepancies were in part motivated by the differences in analysis goals pursued by each method. For instance, although Bambu (Chen et al. 2023b), IsoQuant (Prjibelski et al. 2023), and FLAIR (Tang et al. 2020) rely on the reference annotation to identify transcript models and consequently call few novel transcripts, other tools such as Lyric (Kaur et al. 2024) are designed to detect highly supported novel transcripts. Additionally, LRGASP conducted validation of long-read transcript models both experimentally and by manual curation. Surprisingly, a large number of novel transcripts were confirmed. Many transcripts identified by only one or a few tools were experimentally validated by PCR (Fig. 1B), whereas several novel transcripts that had not been detected by any of the benchmarked tools were confidently annotated by GENCODE (Fig. 1C). Most of these novel transcripts belonged to known genes and included novel combinations of annotated exons, displaced splice donor or acceptor sites, or previously undescribed intron retention events. Moreover, many validated novel transcripts were lowly expressed and found in *single samples* of the same experimental condition.

The LRGASP project highlighted two known but previously underappreciated insights: (i) LRS technologies are also single-molecule sequencing methods that, unlike short-read sequencing, reveal the actual RNA molecules in a biological sample, and (ii) RNA transcription is inherently noisy, and during the transcription and splicing processes, RNA molecules that deviate from the major gene expression products may be synthesized. This results in a pool of rare but real transcripts populating biological samples. Noisiness in RNA synthesis is long known and has been described in association with several stresses (Castells-Roca et al. 2011), diseases (Larsen et al. 2017; Wu et al. 2022), and also as a source of evolutionary adaptation (Singh and Ahi 2022; Wright et al. 2022). These rare transcripts were usually overlooked in conventional short-read sequencing because standard analysis methods interpreted them as sporadic misalignment events and only library preparation techniques designed to target particular transcripts had the resolution to confidently detect lowly expressed transcript variants (Mercer et al. 2014). In contrast, the ability to capture full-length transcripts at ever greater sequencing depths by LRS means these deviations cannot be ignored any longer, presenting both a challenge and an opportunity in transcriptome research.

In this perspective, we discuss two significant consequences of LRS capturing unique, often times sample-specific, real RNA molecules: (i) the need to acknowledge this fraction of the transcriptome as different from the condition-specific transcriptional program and develop bioinformatics methods that distinguish these authentic molecules from technological artifacts to study their biological relevance, and (ii) the considerations of incorporating this transcriptomic complexity into genome annotation efforts. For both issues, we discuss the technology's potential to advance the field, the conceptual shifts it imposes on the research community, and the challenges associated with data processing and analysis.

## Understanding the rare but real transcriptome

**Figure 2.**
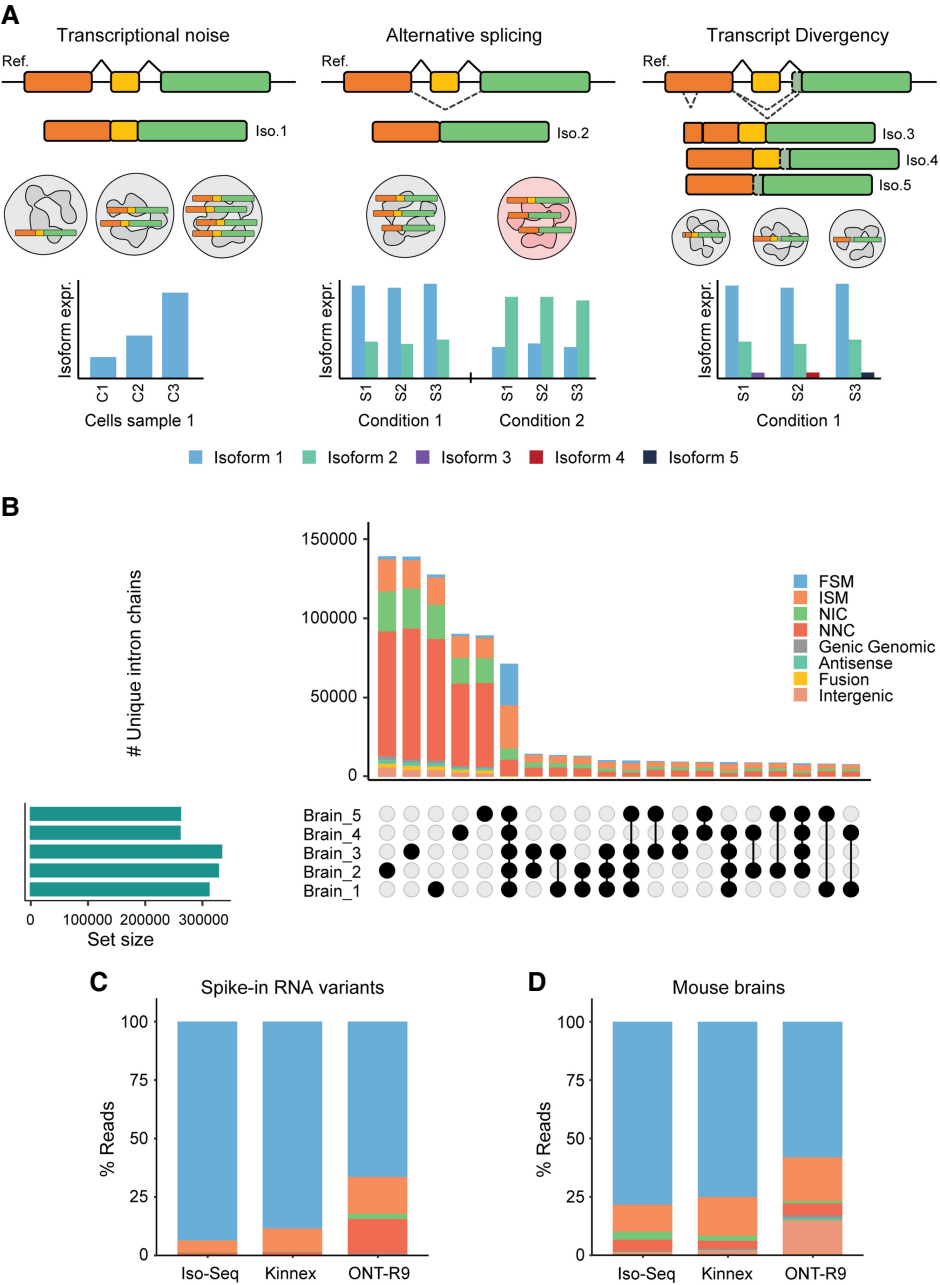
TD detection. (*A*) Differences between TN, alternative splicing (AS), and TD. TN indicates stochastic variation in transcript expression levels. AS is programmed alternative processing of RNA that can be consistently detected. TD represents rare but real RNA molecules. (*B*) Structural category classification and intersection of loci among biological replicates in mouse brain samples. Replication strongly reduces the biological and technical noise in the samples. Structural category classification of reads among PacBio Iso-Seq and Kinnex, and ONT (R9 flowcell) sequencing methods in (*C*) spike-in RNA variants (SIRVs) and (*D*) mouse brains. Non-FSM reads in spikes represent the technical noise level of the technology, whereas the excess of these reads in the real sample is indicative of TD.

We refer to the previously described pool of diverse and rare RNA molecules, expressed alongside their major expression products, as transcript divergency (TD). TD encompasses the stochastic variability in gene expression resulting from deviations in transcription and splicing processes; in other words, it is a population of RNA molecules that diverge from the condition-defining transcriptional state. TD is distinct from transcriptional noise (TN), defined as across-cell variations in expression levels (Angelidis et al. 2019; Tosti et al. 2021; Bartz et al. 2023), and alternative or aberrant splicing, defined as RNA processing characteristic of a cell type or biological condition (Castells-Roca et al. 2011; Larsen et al. 2017; Kahles et al. 2018; Jose et al. 2019). TD molecules are, on the contrary, often times sample-specific and heterogeneous (Fig. 2A).

Why should we care about TD in the first place? An increased transcriptome stochasticity has been described in relation to ageing (Enge et al. 2017; Martinez-Jimenez et al. 2017; Angelidis et al. 2019; Kimmel et al. 2019), disease (Larsen et al. 2017; Wu et al. 2022; Levy et al. 2023; Merlotti et al. 2023; Patowary et al. 2024), and cellular stress in a variety of organisms (Castells-Roca et al. 2011; Nicolas et al. 2018; Wu et al. 2022; Ramnarine et al. 2022). For example, in cancer, misregulated splicing patterns have been proposed as a source of neoantigens that contribute to tumor progression (Levy et al. 2023; Merlotti et al. 2023). In Alzheimer's disease, misregulated alternative splicing (AS) has been observed in genes associated with synaptic plasticity and neuronal function (Larsen et al. 2017; Patowary et al. 2024). As cells age, the accumulation of TN increases errors in gene expression and disrupts pathway coordination, making cells more susceptible to functional decline and age-related conditions (Martinez-Jimenez et al. 2017). TD could contribute to or be a consequence of cellular stress by generating erroneous transcripts or proteins that may interfere with normal cellular functions, potentially leading to an altered phenotype. Moreover, RNA processing deviations from the main transcriptional program may represent a source of transcriptional innovation part of the evolutionary process, and low abundance structural variations may have biological functions, as has been shown for long noncoding RNAs (Mattick et al. 2023). Faithfully identifying these molecules within the LRS signal, will be invaluable to study and characterize these processes and understand how transcriptional stochasticity contributes to transcriptome biology.

Having stated the relevance of TD in biology, the next question is how to analyze and describe this fraction of the transcriptome, and how to differentiate it from technical noise and condition-specific transcript isoforms.

Recent efforts have moved beyond annotating alternative isoforms to conducting in-depth analysis of transcriptional diversity per gene. For instance, Reese et al. (2023) evaluate non-FSM reads based on three diversity mechanisms: TSS, exon junction chain, and TTS, analyzing each gene to determine which of these three diversity mechanisms it is predominantly influenced by Reese et al. (2023). However, structural elements of the RNA molecules are treated as independent variables, despite substantial research on exon exclusion and co-inclusion patterns that highlight the interdependent effects of these elements (Tilgner et al. 2015; Dong and Chen 2020; Oehler et al. 2022; Singh and Ahi 2022; Merlotti et al. 2023). Other tools assess transcriptional diversity through quantification of structural differences and evaluation of distance metrics between transcript models (Nanni et al. 2024). Due to the heterogeneous and stochastic nature of the TD, it is essential to study these molecules comprehensively, considering the full tandem combination of TSS, exon junction chains, and TTS. Therefore, these findings underscore the need for developing new analytical methods that consider transcripts as integrated entities rather than the sum of their parts.

Numerous algorithms that aim at identifying and quantifying transcript models from LRS data have been published (Bushmanova et al. 2019; Kovaka et al. 2019; Wyman et al. 2019; Sahlin and Medvedev 2020; Tang et al. 2020; Holmqvist et al. 2021; Wang et al. 2021; de la Rubia et al. 2022; Gao et al. 2023; Lienhard et al. 2023; Nip et al. 2023; Orabi et al. 2023; Petri and Sahlin 2023; Prjibelski et al. 2023; Volden et al. 2023; Chen et al. 2023b). Generally, they do not make a specific distinction between TD and condition-associated isoforms, but rather between novel and known transcripts or between tissue-specific and ubiquitously expressed transcripts. We advocate for replication as one of the most effective strategies to differentiate TD from regular isoforms. For example, analyzing a high-depth mouse brain lrRNA-seq PacBio Iso-Seq data set (Supplemental Methods), we have observed a bimodal distribution of transcript models across replicates, with most transcripts either present in one sample or in all (5) analyzed samples, with considerably fewer transcripts detected in two to four samples (Fig. 2B). This is relevant as lack of biological replication is still frequent in many LRS studies that favor increased sequencing depth over replication, risking the soundness of the transcriptome composition derived from the LRS data.

A greater challenge, however, is distinguishing between technical and biological noise. A critical, and sometimes overlooked, first step is to run exhaustive quality control analyses on the data. Tools such as LongQC (Fukasawa et al. 2020), SQANTI3 (Pardo-Palacios et al. 2024a), GffCompare (Pertea and Pertea 2020), and SQANTI-reads (Keil et al. 2025) can be used to identify and discard reads and transcript models with common artifacts, such as intrapriming and reverse transcriptase switching. These tools can also be used to identify possible TD molecules, for example, those having noncanonical splicing—splicing events that do not follow the standard *GT-AG* or *GU-AG* splice site patterns. Another important question is the approximate relative magnitude of these two sources of noise, which depends on the chosen LRS method. One way to begin addressing this question is by comparing the amount of deviating reads associated with an invariable set of RNAs, such as those provided by synthetic spike-in RNA variants (SIRVs) or Sequins spike-in controls (Hardwick et al. 2016), to those in a real sample. For example, we used the SQANTI3 framework to analyze the reads associated with Lexogen's E0 mix SIRV transcripts spiked into the aforementioned mouse brain samples, sequenced by different LRS methods, and the reads associated with mouse genes (Fig. 2C,D). Because SIRVs are synthetic known transcripts, all reads should be classified by SQANTI3 as FSM in a technology-error-free scenario. Therefore, the amount of non-FSM reads provides a baseline for the errors associated with the long-read methodology, and any excess of non-FSM reads in the real mouse samples are candidates for TD. We found this difference to be between 10% and 15% of the sequencing output (Fig. 2C).

However, this estimation has several caveats and only represents a possible upper limit. For example, real samples may be subject to higher RNA degradation than the carefully controlled SIRV reagents, so the increased ISM fraction in real samples is likely to represent, at least partly, this additional RNA degradation. Conversely, some native RNAs could be more stable via protection by RNA-binding proteins, tertiary structures, etc. Additionally, SIRV transcript structures are limited in complexity and fall short of faithfully capturing the distribution of transcript length and exon number present in a mammalian transcriptome, being limited for fully recapitulating library preparation and mapping errors. Consequently, for a more precise identification of TD molecules, three efforts should proceed in parallel: (i) continuing to improve LRS and library preparation methods by technology providers, (ii) developing novel, realistic ground-truth standards to differentiate them from any residual technological error, and (iii) developing new and improved isoform and quantification tools capable of identifying and quantifying TD.

To identify reads that represent TD, employing multiple LRS technologies on the same source RNA or utilizing orthogonal data such as short reads, CAGE-seq (Takahashi et al. 2012), and Quant-seq (Moll et al. 2014) can be effective for validating junction sites, TSS, and TTS, respectively. Tools like SQANTI3 are capable of integrating such information to label and filter long-read transcripts, potentially flagging reads that might represent rare RNA species or artifacts. However, this approach is both expensive and may lack generalizability. Moreover, as LRS technologies continue to advance and increase in throughput, their sensitivity for detecting rare junctions, TSS, and TTS will likely surpass that of orthogonal methods, diminishing their utility as supportive evidence. An alternative approach involves statistically modeling RNA processing deviations or learning these patterns from large data sets to train machine learning models capable of classifying them as TD. However, using very strict rules to filter sequencing data sets or training machine learning models on specific sets of data have the potential of discarding real biological TD from less abundant transcripts or those present in sample-specific conditions. Therefore, developing accurate algorithms to identify TD represents a new and important challenge for the computational biology and RNA communities.

## Genome annotation in a context of increasing transcriptional diversity

**Figure 3.**
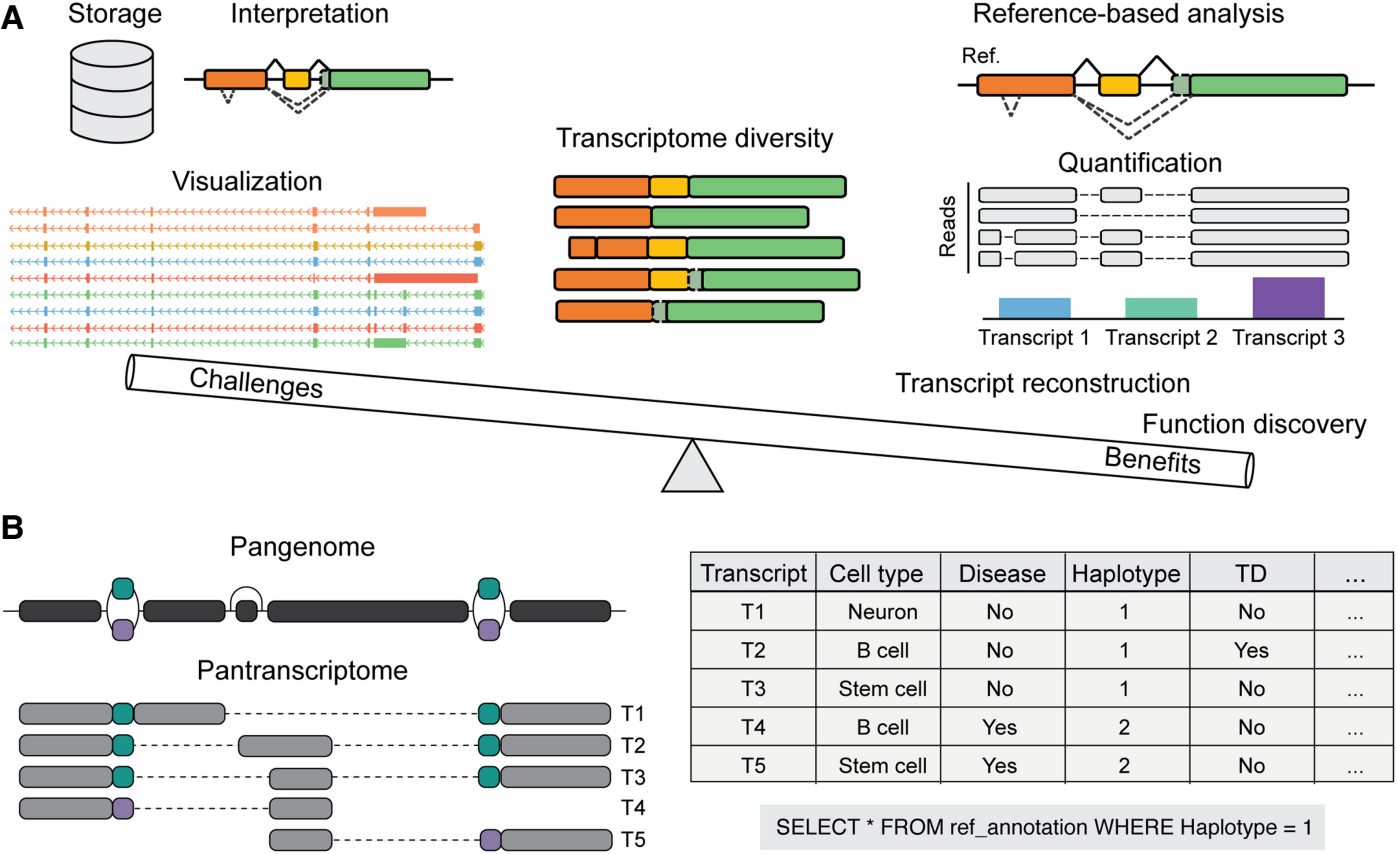
Genome annotation of transcriptome diversity. (*A*) Benefits and challenges of annotating the transcriptome diversity revealed by long-read sequencing methods. Challenges include storage, interpretation, and visualization of vast amounts of transcripts and are outweighed by the benefits of using a comprehensive reference for accurate gene quantification, transcript reconstruction, and function discovery among others. (*B*) Redefining the annotation paradigms. Defining haplotype-specific pantranscriptomes aligns with current pangenome efforts to describe genome diversity. Extensive metadata annotation of the wealth of data provided by LRS allows inclusion and reference customization via subsetting to accommodate diverse analysis scenarios.

The discovery of thousands of transcripts across different species through LRS experiments, once technical and bioinformatic artifacts have been eliminated from the equation, poses a significant challenge for genome annotation. The task is to balance the comprehensive description of this variety with the need to maintain practical and useful reference transcriptomes (Fig. 3A). Effective strategies must be developed to incorporate this complexity without overwhelming the annotation process.

The two reference human gene and transcript annotation resources with the longest standing are NCBI's RefSeq (Maglott et al. 2000; O'Leary et al. 2016) and EMBL-EBI's Ensembl/GENCODE (Harrow et al. 2006; Frankish et al. 2023), both of which started more than 20 years ago. The historic sets of human transcriptomic data that supported the manually annotated transcript models made by both teams consisted of what we would now consider very small numbers of Sanger sequenced expressed sequence tags (ESTs) and cDNAs, with fewer than 0.5 M cDNAs and ∼8.5 M ESTs ever captured. In an era of relative data sparsity, annotating every transcript detected was a plausible goal notwithstanding the immaturity of much of the software and computational tooling supporting the annotation effort. Transcript models based on short-read RNA-seq data have been available for many years, and there is a huge volume of public RNA-seq data on which to base models. Although detailed discussion of RNA-seq methods is out of scope for this perspective, we note that none of the issues described are unique to long transcriptomic methods. For example, with a few years of completion of the human genome sequence, analysis of millions of EST, and cDNA sequences identified splicing complexity (Hayashizaki and Carninci 2006) as did RNA-seq assembly efforts from FANTOM (Hon et al. 2017) and MiTranscriptome (Iyer et al. 2015) a decade later. However, well-understood problems of read length and uncertainty over reliability of assignment of exons and introns to transcript models, particularly in genes with significant evidence of AS have meant that they have not been adopted by Ensembl/GENCODE as part of the reference annotation, and sets produced by RefSeq (tagged as XM, XR, XP), are released as an adjunct to manually annotated models (NM, NR, NP) and not subject to further manual curation (O'Leary et al. 2016). Today, decades-worth of our original endeavors to sequence full-length transcripts can be overtaken by a single PacBio Kinnex or ONT cDNA/directRNA sequencing experiment. The depth of data now available challenges both our technical ability to identify and describe every transcript and our philosophical approach to producing reference annotation. Put simply, sampling depth will increase and many novel transcripts will be captured by reads at a quality that will allow them to be accurately mapped and this has the potential to massively increase the number of transcripts that can be added to reference transcript sets like GENCODE, perhaps by many millions. Increasing the depth of long-read sequencing will also improve the detection of other RNA species such as circular RNAs (circRNAs), single-stranded covalently closed loops of RNA formed by an AS pathway. CircRNA detection requires the use of specific library preparation protocols, but these have been developed for both ONT and PacBio sequencing platforms (You et al. 2015; Rahimi et al. 2021). CircRNAs also present challenges to representation in standard linear genome browsers to identify their distinct biology compared to linear RNA species. An analogous challenge applies to intragenic *trans*-spliced RNAs, where a transcript contains noncollinear splicing, e.g., exons may be spliced out of order. *Trans*-spliced RNAs will be sequenced in long transcriptomic experiments but are likely to be classified incorrectly without specific mapping strategies (Chen et al. 2023a). How should reference annotation resources approach this challenge?

Reference annotation resources must provide their users with information about genes and transcripts that support the downstream analysis they are undertaking. Different users will want to perform different downstream analyses that may benefit from using different transcript annotations, which may appear incompatible. For example, when using RNA-seq data to perform gene-level transcriptomic analysis, a maximal representation of transcriptomic complexity is beneficial to ensure that as many RNA-seq reads as possible are correctly assigned to their gene of origin. This would require that the annotation captures the maximal extent of the gene even where there is variability in the transcript start and end through the use of alternative TSS and TTS sites, also capturing all AS events and potentially even intron retention. Similarly, LRGASP demonstrated the benefit of a comprehensive reference annotation, as reference-guided transcript annotation tools were able to accurately identify many more transcripts than those methods that did not use reference annotations (Pardo-Palacios et al. 2024b). It is reasonable to assume that a complete reference annotation that captures every observed transcript would support reference-guided annotation methods to maximize read mapping.

A further benefit of comprehensive reference annotation is highlighting features that may potentially be overlooked in the absence of annotation. For example, “deep intronic” variants—genetic variants that are more than 100 bp away from the closest exon–intron boundary—may not be identified by standard variant annotation pipelines, or if identified may not be considered significant. Comprehensive annotation that captures infrequently included exons and splice sites may highlight possible functional significance of these previously un- or under-annotated variants. Such variation in exonic and intronic splice enhancing or splice silencing signals may affect the inclusion rate of the exon, and by upregulating the inclusion of the exon in more transcripts from a haploinsufficient gene, could be implicated in disease by disrupting the amount of functional transcripts and the protein they encode.

Conversely, for other applications, a more minimal representation of the transcriptional output of the locus is beneficial, where many transcripts are annotated at a gene-capturing alternative TSS, poly(A) sites, and splicing, this may lead to the annotation of multiple alternative coding sequences (CDSs), perhaps with multiple translation initiation sites and termini. This can be problematic for the annotation/interpretation of variation data where the base(s) affected by a variant may have multiple possible functional consequences assigned. The same genomic position can be assigned as affecting a CDS, 5′ or 3′ UTR, core or proximal splice site sequence or intronic sequence depending on the number and characteristics of the transcripts annotated at a gene. Indeed, the annotation of multiple CDSs in different frames could lead to the same variant being called as synonymous, nonsynonymous or as a loss-of-function variant (Frankish et al. 2015).

Similarly, although a comprehensive annotation would be beneficial for transcript identification and gene-level quantification, it may instead hinder accurate isoform-level quantification by causing over-dispersion of counts between structurally similar isoforms, or even assigning reads to rare isoforms not expressed in the analyzed sample. Therefore, using as references a minimal representation of transcriptional output and personal genome sequences may yield more accurate quantification results for isoform-level quantification.

Another practical consideration of capturing large numbers of transcripts is the effect it may have on visualizing genomic data. Genome browsers such as Ensembl (Martin et al. 2023) and UCSC (Nassar et al. 2023) are frequently the primary access point for interrogating the intersection between genomic data and reference annotation, including gene and transcript annotation. Significant inflation in the number of transcripts annotated at a locus can negatively affect the browsing experience even where compression options are available. Even using browsers like IsoVis (Wan et al. 2024), specifically developed to deal with high amounts of isoforms, may eventually run into the same issues. In simple terms, where there are many transcripts it reduces the space on the screen to display other data tracks and can also make interpretation more difficult as tracking relationships between features may be affected by the increased space between them and the presence of interposed transcript models.

Our ability to describe a transcriptome has historically been constrained by the requirement to map all our transcriptomic data to a reference genome. However, such reference genomes have many loci that do not accurately represent actual transcriptional output on the reference allele or haplotype. To alleviate reference bias effects, high-quality sequences are being generated for pangenome projects for many species; for example, the human reference pangenome project (∼1200 human genomes) (Liao et al. 2023), with some at telomere-to-telomere quality (Schneider et al. 2017) as well as the model organisms mouse (Keane et al. 2011), rat (de Jong et al. 2024), farmed animals such as cow (Smith et al. 2023) and pig (Miao et al. 2024) and the crop plants rice (Shang et al. 2022) and brassicas (Golicz et al. 2016). These pangenome resources promise to revolutionize our ability to accurately map transcriptomic data to the haplotype from which it originates, supporting the confident identification of expression and splicing QTLs and allowing the creation of haplotype-specific representation of the transcriptome. Methods have already been developed to map RNA-seq data to pangenome graphs e.g., VG and RPVG (Sibbesen et al. 2023), and HISAT2 (Kim et al. 2019). These methods demonstrate the improvements in alignment achieved by using the graph over a single reference genome, and they are planned to be extended to support the mapping of lrRNA-seq data. This is likely to reveal haplotype-specific or haplotype-enriched or depleted splicing and transcripts. This presents a further challenge to the groups producing reference gene and transcript annotation. It will be necessary to maintain and improve the annotation of the reference genomes that are likely to remain very widely used for the foreseeable future but also to produce the haplotype-specific annotation that will be needed to harness the full potential of the pangenome. Currently, new haplotypes are frequently annotated by mapping or projecting annotation from the reference genome, with some de novo annotation to add genes, but as transcriptomic data sets can be mapped to genetically identical (or at least very closely related) haplotypes it will be essential to record haploytpe-specific transcripts. As the pangenome and its associated pantranscriptome matures, it will become both possible and necessary to accurately reflect haplotype-specific splicing in the reference gene and transcript annotation produced for the pangenome, but at least initially the annotation on the pangenome will have to contend with some of the challenges for annotation of transcripts on a single reference genome. Comprehensive annotation of all possible transcripts that could be considered TD has the potential to add large numbers of transcripts that are not relevant to the reference genome and many other haplotypes (and could be considered false positive errors in these haplotypic contexts), however, excluding transcripts from the set would lead to their subsequent exclusion (and false negative errors) when annotation from a single reference genome is projected to alternative haplotypes.

A reference annotation can take one of two broad approaches on how to deal with TD (it should always seek to exclude technical artifacts). Reference annotation can be inclusive and capture transcripts comprehensively or it can seek to exclude transcripts defined as TD. Although this latter approach is both attractive and technically feasible, it presupposes that we have sufficient scientific knowledge and resolution in the data to exclude all biological noise and include all “real” transcripts. An exclusionary approach may be able to achieve something quite close to an ideal minimal representation of transcripts, but it may not be future-proof to new understanding of genome biology, developments in experimental methods and data generation and computational tools. We can take a lesson from the historical annotation produced by the predecessor to Ensembl/GENCODE in the era of data scarcity at the time the original sequencing of the human genome was completed (Benson et al. 1997). At this time, although much attention was paid to the annotation of protein-coding transcripts, transcripts with premature termination codons likely to be subject to nonsense-mediated decay (NMD) were also annotated along with transcripts that retained intronic sequence. A comprehensive annotation of pseudogenes of protein-coding genes was also produced and transcripts with no obvious protein-coding potential were also annotated. In all these cases, transcript features were recorded in the annotation without a clear understanding of their relevance to the function of the cell and in all cases it can be argued that their annotation was well founded and proved useful. NMD in particular but also intron retention have been demonstrated as important in posttranscriptional gene regulation (Jacob and Smith 2017; Monteuuis et al. 2019), comprehensive pseudogene annotation has supported analysis of genome evolution and been practically useful in understanding mapping queries in more recent transcriptomic data (Frankish and Harrow 2014; Amaral et al. 2023), and the noncoding transcripts anticipated the then-nascent, now vast field of long noncoding RNA study (Mattick and Rinn 2015; Ramilowski et al. 2020). Not throwing data away but capturing and labeling it as well as possible has proved useful since the original annotation efforts. Even where transcript structures remain unchanged over the years (because the original decision of the starts and ends of exons was correctly determined early on), broader and deeper annotation and metadata may be layered on to the original models. This cannot happen if the transcript is not part of the annotation.

Given this, a goal for reference annotation should be to capture everything, every transcript structure and CDS, every TSS and polyadenylation site, and do it in a haplotype-specific way across the pangenome (Fig. 3B). Data generation methods that are currently available, along with improved computational methods will support movement toward this goal. If the transcript set is maximally inclusive, to support the broadest possible variety of analysis by downstream users then it must be maximally labeled to support the filtering or subsetting of the larger set to provide the transcripts that are best suited for a users specific analysis.

Simple subsets of transcripts already exist in reference annotation. For example, GENCODE has several sets of transcripts readily identifiable in the genome browsers and release files; GENCODE comprehensive (everything), GENCODE basic (subset of full-length coding transcripts and minimal representation of other gene biotypes), GENCODE primary (smaller subset of transcripts of likely functional significance based on expression and evolutionary conservation and constraint) (Frankish et al. 2023), MANE Select, MANE Plus Clinical (subsets of transcripts agreed with NCBI RefSeq based on expression, evolutionary conservation and constraint, developed to support the consistent reporting of clinical variation) (Morales et al. 2022), APPRIS principal isoforms (protein-centric analysis to determine likely functional isoforms) (Rodriguez et al. 2018). These somewhat naive subsets are useful in providing an initial set of transcripts for analysis, browser display and variant reporting but they point to the beginning of the possibility rather than an end point.

As the number of reads generated by lrRNA-seq experiments explodes, allowing us to detect many transcripts including those that are very rare, haplotype-specific, cell-state or stimulation specific, etc., we will also have greater power to describe every transcript added to reference annotation. Every annotated transcript can be compared to reads from any sequencing experiment deposited in a public sequence archive to determine whether its expression was detected in that experiment, and if it was, extract what were the absolute and relative expression values in terms of reads or proportion of transcription from its locus represented by the transcript. In addition, the lack of detection of the transcript in an experiment may also be recorded. In future, analysis pipelines will be able to identify the most appropriate haplotype in the reference pangenome, for mapping to ensure haplotype-specific transcripts are appropriately placed. These data can be accurately captured for every transcript, alongside metadata from the experiment such as tissue, cell type, activation state, developmental stage, age, sex, disease state, etc. to be stored in a database (Fig. 3B). The database could be updated with new transcript annotation and experimental data as they become available and permit customization based on any captured metadata (e.g., tissue and abundance) allowing users flexibility to create the right data set for their analysis or create automated subsets based on predetermined filter parameters. Integration with genome browsers might allow the fly creation of annotation tracks for display, whereas archiving strategies and detailed filterset metadata could be included to allow the recreation of past filtered transcript sets at any time.

## Concluding remarks

Single-molecule LRS technologies have demonstrated an unprecedented capacity to uncover the vast diversity of both common and rare RNA molecules that constitute the transcriptomes. Traditionally, data analysis methods have focused on identifying and characterizing the consistent and functional components of the transcriptome. However, the wealth of new data from these advanced technologies suggests that rare but genuine transcripts can no longer be ignored, but at the same time, not every single new transcript found in an LRS experiment may require the same consideration. Embracing this potential necessitates the development of new analysis methods and a redefinition of existing paradigms. Innovative analytical procedures are required to effectively distinguish technical artifacts from biological noise and to assess their biological relevance in a variety of contexts. Concurrently, new strategies and protocols must be established to annotate the ever-growing diversity of transcriptomes in a manner that is useful for both current and future research. Rather than simplifying analysis, LRS presents exciting analytical challenges, demanding sophisticated approaches to manage the vast expanse of molecular data being discovered.

## Data access

All data generated in this study have been submitted to the European Nucleotide Archive (ENA; https://www.ebi.ac.uk/ena/browser/home) under accession number PRJEB85167.

## Supplemental Material

Supplement 1
