# Supplementary material for: Notable challenges posed by long-read sequencing for the study of transcriptional diversity and genome annotation
Source: Genome Res. 2025 Apr;35(4):583–92. doi: 10.1101/gr.279865.124 (PMC12047247; doi:10.1101/gr.279865.124)
Supplement: Supplement 1 [file Supplemental_Methods.docx]

**Supplementary methods**

**Animal Care and Brain Collection**Five male C57BL6/J wild-type mice (Jackson Laboratories) were housed in groups with access to standard pelleted food and tap water *ad libitum* at the University of Valencia’s animal housing facility (Servei Central de Suport a la Investigació Experimental – SCSIE, Burjassot). At 3 months of age, mice were euthanized by cervical dislocation. Brains were rapidly collected, snap-frozen in liquid nitrogen, and stored at −80°C. All procedures adhered to European Union (86/609/EEC) and Spanish (RD1201/2005) regulations, with protocol approval from the Ethics Committee on Experimental Research of the University of Valencia.

**RNA Extraction**Brain tissues were homogenized using a FastPrep system (MPBio) with 1.4 mm zirconium spheres. Total RNA was extracted using Maxwell® 16 LEV simplyRNA Purification Kits (Promega) on an automated Maxwell16 device. RNA quality and quantity were assessed with a Qubit RNA BR Assay Kit (ThermoFisher Scientific) and an Agilent TapeStation System (Agilent Technologies). Lexogen SIRV-Set1 (subset E0) spike-ins were added according to the manufacturer’s protocol.

**PacBio Iso-Seq and Kinnex Library Preparation, Sequencing and data processing**For Iso-Seq, cDNA synthesis and barcoding were performed with the NEBNext Single Cell/Low Input cDNA Synthesis and Amplification Kit, following the manufacturer’s instructions. Samples were pooled into two groups (three and two brains per pool) and sequenced using the PacBio IsoSeq SMRTbell prep kit 3.0. Each pool was sequenced over eight runs on a PacBio Sequel IIe at SCSIE, generating an average of 4.36 million reads per sample.
For Kinnex sequencing [(Al’Khafaji et al. 2024)](https://paperpile.com/c/EaA29F/Re6A), cDNA synthesis, barcoding, and library preparation were conducted using the Kinnex full-length RNA kit (Pacific Biosciences) and sequenced on a PacBio Revio system. Segmented reads were split using Skera (https://skera.how/), yielding an average of 41.8 million reads per sample.
For both datasets, reads were demultiplexed and primers removed with Lima (v2.9.0) (https://lima.how/). Poly(A) tails and concatemers were processed using Isoseq refine (https://github.com/pacificbiosciences/isoseq/). BAM files were converted to FASTQ format with Bamtools (http://github.com/pezmaster31/bamtools), and reads were aligned to the mm39 NCBI RefSeq genome using minimap2 (v2.17) [(Li 2018)](https://paperpile.com/c/EaA29F/XuFD) with the parameters -ax splice:hq -uf.

**Nanopore Library Preparation, Sequencing and data processing**cDNA synthesis and barcoding were conducted using the Nanopore SQK-PCB111-24 PCR-cDNA Barcoding Kit, following standard protocols. Eight libraries per sample were generated, pooled, and sequenced across eight runs on R9 flow cells (FLO-PRO002) using a PromethION 2 platform at LongSeq applications, yielding a mean of 3.5 milion reads per sample.
Raw Pod5 files were processed with Dorado (v7.2.13, High-accuracy model, 450 bp) (https://github.com/nanoporetech/dorado). Read quality was verified with FastQC (v0.12.0) (https://www.bioinformatics.babraham.ac.uk/projects/fastqc/), ensuring a Phred score above 20. FASTQ files were merged and stranded with Pychopper (v2.5.0) (https://github.com/epi2me-labs/pychopper). Full-length transcripts were aligned to the mm39 NCBI RefSeq genome using minimap2 (v2.17) [(Li 2018)](https://paperpile.com/c/EaA29F/XuFD) with the parameters -ax splice -uf.

[Al’Khafaji AM, Smith JT, Garimella KV, Babadi M, Popic V, Sade-Feldman M, Gatzen M, Sarkizova S, Schwartz MA, Blaum EM, et al. 2024. High-throughput RNA isoform sequencing using programmed cDNA concatenation. *Nat Biotechnol* **42**: 582–586.](http://paperpile.com/b/EaA29F/Re6A)

[Li H. 2018. Minimap2: pairwise alignment for nucleotide sequences. *Bioinformatics* **34**: 3094–3100.](http://paperpile.com/b/EaA29F/XuFD)
